# Ewha, where medical education for women began in Korea, is now leading global healthcare through innovative research networks and education: the inaugural address of the 28th Dean of the College of Medicine

**DOI:** 10.12771/emj.2025.00129

**Published:** 2025-03-26

**Authors:** Duk-Hee Kang

**Affiliations:** Division of Nephrology, Department of Internal Medicine, College of Medicine, Ewha Womans University, Seoul, Korea

Dear beloved students, respected faculty, and esteemed alumnae and staff who uphold Ewha’s cherished traditions and values,

I am honored and privileged to greet you as the 28th Dean of Ewha Womans University College of Medicine (Fig. 1). Communicating with you in this message fills me with both joy and a profound sense of responsibility.

Ewha Womans University College of Medicine holds a unique position as Korea’s pioneering institution for women’s medical education. Reflecting on our history, I recall the selfless devotion of women medical missionaries who, 138 years ago, journeyed to the unfamiliar shores of Joseon, established the Bogu Yeogwan (普救女館), and dedicated themselves to education, healthcare, and missionary work. Although being the “first” is historically significant, the enduring spirit and core values upon which Ewha was founded—spreading God’s love, serving those in need, and striving to create a better society through education—are even more valuable.

There are things whose value diminishes over time, such as new buildings, advanced facilities, and premium products. In contrast, cultivating exceptional medical professionals, conducting groundbreaking research that seeds future medicine, and accumulating unparalleled expertise and experience grow ever more valuable with time. These are the essential values that Ewha must continuously uphold.

Our nation’s healthcare system now faces significant challenges. The once-envied medical system is now confronted with doubts about its sustainability. Numerous issues await resolution, including disruptions and temporary delays in medical education, ensuring an adequate healthcare workforce, and addressing critical gaps in essential medical care. Even amid unprecedented pressures that keep clinical professors working tirelessly around the clock in clinics and wards, the members of Ewha Womans University College of Medicine have chosen not to merely lament or complain. Instead, we have remained steadfast in our mission by sustaining diverse educational initiatives and research support for our faculty—even during the temporary suspension of formal medical education in 2024.

As the newly appointed Dean, I present the following strategic objectives to propel Ewha Womans University College of Medicine into a new era of advancement (Fig. 2):

First, we aim to build a sustainable Ewha medical education system that is resilient to challenges.

Second, we seek to enhance faculty research capabilities through tailored support and by fostering a collaborative research ecosystem.

Third, we intend to cultivate Ewha’s global medical leadership by expanding and solidifying high-level international exchanges.

Fourth, we must recognize that significant progress cannot be achieved solely by the efforts of a few individuals. To nurture a collaborative Ewha medical community founded on open communication, mutual understanding, and cooperation, we aim to establish a culture of respect and appreciation among staff, researchers, and faculty, empowering everyone to contribute confidently and with pride. Above all, we commit to sincerely listening to and acting on the valuable insights of our alumnae, whose unwavering trust and affection guide us.

Ewha Womans University College of Medicine owes its current success to each of you. Our continued growth and future achievements depend on your unwavering support. I earnestly hope that Ewha, long renowned for pioneering women’s education in medicine, will truly embody the biblical call to “Arise, shine, for your light has come, and the glory of the LORD has risen upon you” (Isaiah 60:1), thereby illuminating our era.

To our beloved students, who may be facing particularly challenging times, I offer these words: our studies and research are not intended to construct ivory towers isolated from society’s realities but rather to embark on a journey toward discovering the most effective ways to heal those we encounter daily. Despite temporary setbacks and delays, remain steadfast to the “grand aspirations” with which you entered Ewha and the “excellent habits” you have diligently cultivated. Together, these will serve as an unwavering shield against life’s uncertainties.

The doors of the Dean’s office remain open at all times. I invite you to share your thoughts, encouragement, and hopes for Ewha’s continued ascent.

Thank you.

## Figures and Tables

**Fig. 1.** Duk-Hee Kang. The 28th Dean of the College of Medicine at Ewha Womans University.

**Fig. 2.**
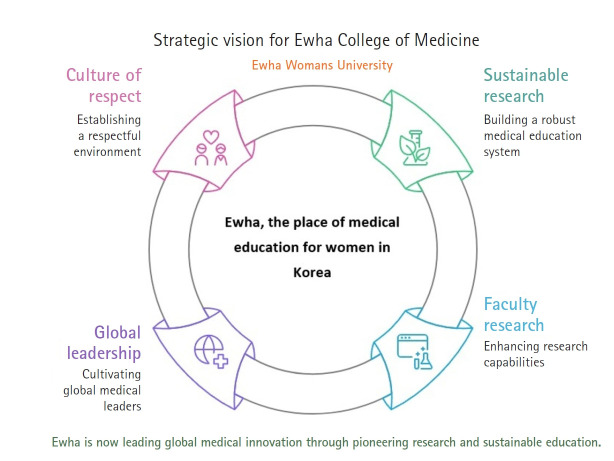
Four strategic objectives to propel Ewha College of Medicine into a new era of advancement.

